# Assessment of Aflatoxin Contamination of Maize, Peanut Meal and Poultry Feed Mixtures from Different Agroecological Zones in Cameroon

**DOI:** 10.3390/toxins5050884

**Published:** 2013-04-29

**Authors:** Jean Raphaël Kana, Benoit Gbemenou Joselin Gnonlonfin, Jagger Harvey, James Wainaina, Immaculate Wanjuki, Robert A. Skilton, Alexis Teguia

**Affiliations:** 1 Animal Production Laboratory, Department of Animal Science, University of Dschang, Cameroon; 2 Biosciences eastern and central Africa-International Livestock Research Institute (BecA-ILRI) Hub, Nairobi, Kenya

**Keywords:** aflatoxin occurrence, feedstuffs, animal feed, broilers, layers, poultry, Cameroon

## Abstract

Mycotoxins affect poultry production by being present in the feed and directly causing a negative impact on bird performance. Carry-over rates of mycotoxins in animal products are, in general, small (except for aflatoxins in milk and eggs) therefore representing a small source of mycotoxins for humans. Mycotoxins present directly in human food represent a much higher risk. The contamination of poultry feed by aflatoxins was determined as a first assessment of this risk in Cameroon. A total of 201 samples of maize, peanut meal, broiler and layer feeds were collected directly at poultry farms, poultry production sites and poultry feed dealers in three agroecological zones (AEZs) of Cameroon and analyzed for moisture content and aflatoxin levels. The results indicate that the mean of the moisture content of maize (14.1%) was significantly (*P* < 0.05) higher than all other commodities (10.0%–12.7%). Approximately 9% of maize samples were positive for aflatoxin, with concentrations overall ranging from <2 to 42 µg/kg. Most of the samples of peanut meal (100%), broiler (93.3%) and layer feeds (83.0%) were positive with concentrations of positive samples ranging from 39 to 950 µg/kg for peanut meal, 2 to 52 µg/kg for broiler feed and 2 to 23 µg/kg for layer feed. The aflatoxin content of layer feed did not vary by AEZ, while the highest (16.8 µg/kg) and the lowest (8.2 µg/kg) aflatoxin content of broiler feed were respectively recorded in Western High Plateau and in Rainforest agroecological zones. These results suggest that peanut meal is likely to be a high risk feed, and further investigation is needed to guide promotion of safe feeds for poultry in Cameroon.

## 1. Introduction

Food security remains an important Millennium Development Goal in many developing countries. Poultry production is one of the routes being used to achieve food security. Since the total ban on importation of poultry products in Cameroon in 2001, the poultry industry has become one of the most important animal husbandry sectors, achieving impressive growth in the last 10 years. In Cameroon and in the whole central Africa region, environmental conditions and inadequate storage practices provide optimal conditions for fungal growth and mycotoxin accumulation in foods and feedstuffs [1]. The incorporation of various ingredients of plant origin into poultry feed mixtures increases the risk of contamination by fungi and their toxic metabolites [2,3]. Aflatoxins are a major concern to poultry production and health because of serious economic losses [3,4]. Aflatoxin B_1,_ is the most potent natural carcinogen known [5] and may pass to poultry products, such as meat or eggs at very low levels [3]. Recent studies have demonstrated that aflatoxin exposure through a variety of sources is linked with growth impairment in animals and humans [3,5,6,7]. Aflatoxins are liver toxins and especially B_1_, are recognized as inhibitors of nucleic acid and protein synthesis in animals [8]. Aflatoxicosis in poultry also causes anorexia with lowered growth rate, poor feed utilization, decreased egg production and increased mortality [3,7,9]. It also modifies lipid metabolism and the mitochondrial respiratory pathway, where an excessive accumulation of lipids may be noticed in the liver. Additionally, anemia, reduction of immune function, hepatotoxicosis, hemorrhage, teratogenesis, carcinogenesis and mutagenesis are associated with aflatoxicosis [10,11]. 

Rodrigues *et al.* [2] recently conducted a survey of the occurrence of mycotoxins in feedstuffs and finished feeds in the Middle East and Africa, which included numerous samples from Western and Central Africa including Nigeria, Sudan, Egypt, Algeria, Kenya, Ghana, South Africa, Israel, Jordan, Lebanon, Syria and Yemen. They found that 98% of the ingredients used in animal feed formulation are positive for aflatoxin B_1_. They also showed that maize is a preferred substrate for fungal growth and mycotoxin production in comparison with soybean and wheat. However, no samples were taken from Cameroon, which borders Nigeria. In Cameroon, food commodities are highly susceptible to fungal infections that tend to increase with length of storage [1,12]. Generally in this country, moldy grains end up as animal feeds and there is no information about the levels of aflatoxin or on the risk of significant animal exposure. One of the key determinants of aflatoxin accumulation in maize, peanuts and other crops is moisture content [1]. 

Poultry feed in Cameroon typically consists of maize, peanut meal (residue after extraction of oil for human consumption), and different mixes of maize, soybeans and other crops. The aim of the present study was to evaluate the occurrence of aflatoxins in poultry feeds, including peanut meal and maize. This was determined across three AEZs of Cameroon: Sahelian zone, Western High Plateau and Rainforest that account for approximately 90% of the poultry farms in the country and a high amount of maize production.

## 2. Material and Methods

### 2.1. Agroecological Zones

**Figure 1:**
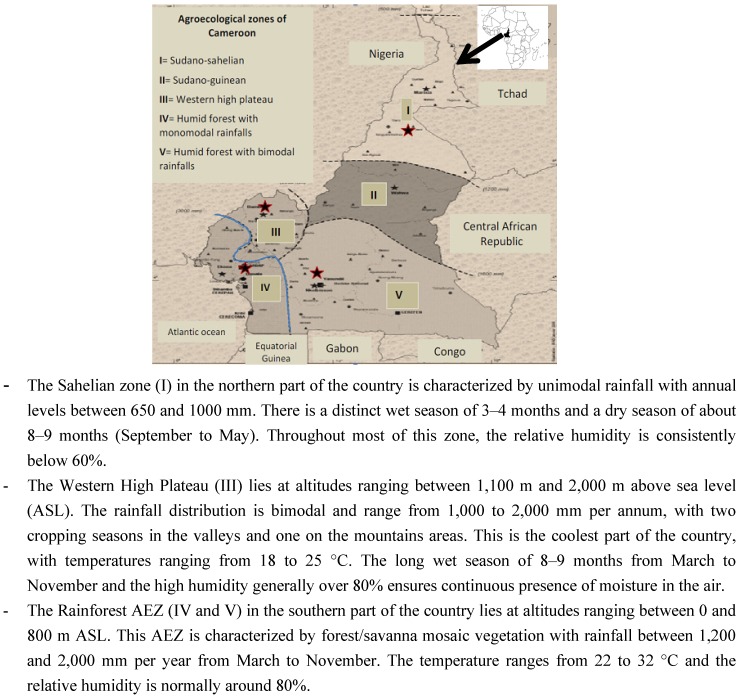
Sampling sites across different agroecological zones of Cameroon.

Cameroon consists of five major AEZs that include: Sudano-Sahelian (I) in the north and extreme north region, Sudano-guinea (II) in the Adamaoua Plateau, Western High Plateau (III) in West and North-west region, Humid Forest with unimodal rainfalls (IV) in the Littoral and Southwest region, and the Humid Forest with bimodal rainfalls (V) in Central and Eastern part of the country. In this study, samples were collected in three AEZs selected according to their importance in maize and poultry production in the country (Figure 1).

### 2.2. Sampling

Between May and August 2012, a total of 201 samples of feedstuffs and poultry feeds (41 samples of peanut meal, 30 samples of broiler feed, 53 samples of layer feed and 77 samples of maize) were randomly collected directly from smallholder poultry farms, poultry feed production sites or from poultry feed dealers in the three AEZs of Cameroon as described above. Poultry feeds and peanut meal were collected in Bafoussam, Dschang and Bamenda in Western High Plateau AEZ, and in Yaoundé and Douala in the Rainforest AEZ. These regions were selected because they have the largest proportion (~90%) of poultry farms in Cameroon. During the same period, maize samples (37 samples of white and 40 samples of yellow maize) were collected in Western High Plateau and Sahelian zones in the northern part of the country. These AEZs were chosen based on their significance in terms of maize production. The samples were stored in plastics sacks at room temperature (20–25 °C) until they were analyzed in September 2012; all samples were sealed under vacuum to prevent air exchanges between the samples and the storage environment.

### 2.3. Determination of Moisture Content

Moisture content of samples was determined using the standard oven method [13]. The samples were weighed, dried in duplicate at 100 °C to constant weight and the mean moisture content was calculated on a percentage dry basis.

### 2.4. Determination of Aflatoxin Content

#### 2.4.1. Aflatoxin Extraction

All the samples were ground using a Romer Mill (Romer series II^®^ MILL), and from each sample 5.0 g was weighed, mixed with 0.5 g sodium chloride. 10 mL of 80% methanol solution (methanol:water, 80:20 v/v) was added and mixed at 225 rpm for 4 min at 25 °C in a controlled environment shaker (New Brunswick CO. INC, EDISON, N.J., USA). The mixture was filtered using fluted filter paper (Folder Grade: 1289, VICAM, A Waters Business), 2 mL of filtrate was diluted with 8 mL of distilled water in a clean tube, and mixed for 2 min on a Denley Spiramix linear mixer (Denley, Sussex, UK). Two mL of diluted filtrate (0.2 g sample equivalent) was passed through an Aflatest^®^-P affinity column at a rate of 1 to 2 drops/second and the column was then rinsed twice with 5 mL of distilled water at the same rate. The aflatoxin material bound to the affinity column was eluted with 1.0 mL HPLC grade methanol at the rate of 1 to 2 drops/second and the eluate was collected in a glass tube. 

#### 2.4.2. Aflatoxin Quantification

One mL of Aflatest^® ^developer solution was added to the eluate from the Aflatest-P column, mixed, and concentrations of total aflatoxin (B1 + B2 + G1 + G2) (µg/kg) were detected after 60 seconds using a Vicam fluorometer (Series-4EX, Source Scientific LLC, USA) calibrated with a blank of methanol, with the standard manufacturer’s protocol. The detection limit of this method was 2.0 µg/kg and upper limit was 300 µg/kg. For samples above 300 µg/kg, extracts were further diluted an additional X5 for an upper limit of 1500 µg/kg.

### 2.5. Data Analysis

Data were summarized and analyzed using SPSS (version 12.0) and Duncan’s multiple range was used to determine differences in the means among samples obtained from the different AEZs (*P* = 0.05).

## 3. Results

### 3.1. Moisture Content

One of the predisposing factors for aflatoxin accumulation in maize, groundnut and other crops after harvest is moisture content. Mean moisture content was higher (*P* < 0.05) in maize (14.1%) compared to all other commodities (10.0%–12.7%) with peanut meal having the lowest levels (10.0%) (Table 1). In Western High Plateau, moisture content of maize samples ranged from 11.7% to 17.0% with an average of 13.7%, while in Sahelian zone it range from 12.9% to 17.7% with an average of 15.4%. In peanut meal, the average of moisture content was 10.1 and 9.8% respectively in Western High Plateau and Rainforest zone. In poultry feeds, the extremes of moisture content were recorded in Rainforest with an average of 10.7% in broiler feed compared to 12.7% recorded in layer feed. 

**Table 1:** Moisture content of maize, peanut meal and poultry feed mixtures in different agroecological zones of Cameroon.

Commodities	Agroecological zones	N	Range (%)	Mean within the agro-zone (%)	Mean (%)	SEM
Maize (N = 77)	Western High Plateau	62	11.7–17.0	13.7	14.1^a^	0.8
	Sudano/Sahelian	15	12.9–17.7	15.4		
Peanut meal (N = 41)	Western High Plateau	33	8.5–11.8	10.1	10.0^d^	0.1
	Rainforest	8	9.1–10.5	9.8		
Broilers feed (N = 30)	Western High Plateau	13	9.9–14.7	12.2	11.3^c^	0.3
	Rainforest	17	8.4–17.0	10.7		
Layers feed (N = 53)	Western High Plateau	33	10.2–16.1	12.6	12.7^b^	0.2
	Rainforest	20	10.8–15.3	12.7		

^a^^, b, c, d ^Means within a column with different superscripts are different (*P* < 0.05).

### 3.2. Aflatoxin Contamination

Aflatoxin levels in the four feed categories were assessed. All peanut meal contained aflatoxin above the limit of detection of the method (2 µg/kg). In 9.1% (7/77) of maize aflatoxin concentrations ranged from 2 to 42 µg/kg. Overall, 57.1% (4/7) of positive maize samples contained less than 5 µg/kg of aflatoxin while 42.9% (3/7) contained more than 5 µg/kg. Broiler feeds (11.2 µg/kg) showed approximately two times higher levels of aflatoxin compared to layer feeds (6.6 µg/kg). Similarly, 35.7% (10/28) of broiler feed samples contained more than 10 µg/kg of aflatoxin compared to 22.7% (10/44) of layer feeds (Table 2). Overall in feeds (broiler and layer), 87% were positive for aflatoxin (72/83).

**Table 2:** Aflatoxin level in maize, peanut meal and poultry feed mixtures in Cameroon.

Commodities	Aflatoxin occurence	Range (µg/kg) (Min–Max)	Mean (µg/kg)	SEM	Frequency (of positives)	
Maize (N = 77)	7/77 (9.1%)	≤2–42 µg/kg	1.0^a^	0.5	<5 µg/kg	4/7 (57.1%)
					>5µg/kg	3/7 (42.9%)
Peanut meal (N = 41)	41/41 (100%)	39–950 µg/kg	161.4^b^	27.5	<50 µg/kg	4/41 (9.8%)
					50–100 µg/kg	21/41(51.2%)
					>100 µg/kg	16/41 (39.0%)
Broiler feeds (N = 30)	28/30 (93.3%)	<2–52 µg/kg	11.1^a^	2.2	<5 µg/kg	8/28 (28.6%)
					5–10 µg/kg	10/28 (35.7%)
					>10 µg/kg	10/28 (35.7%)
Layer feeds (N = 53)	44/53 (83.0%)	≤2–23 µg/kg	6.6^a^	0.7	<5 µg/kg	15/44 (34.1%)
					5–10 µg/kg	19/44 (43.2%)
					>10 µg/kg	10/44 (22.7%)

^a^^, b ^Means within a column with different superscripts are different (*P* < 0.05).

**Figure 2:**
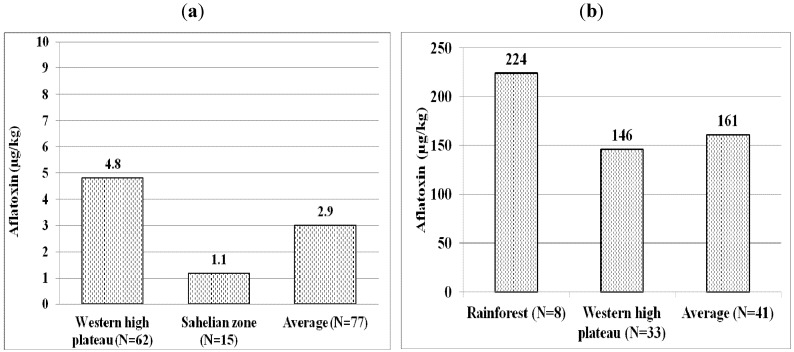
(**a**). Average aflatoxin content of aflatoxin positive maize samples. (**b**). Average aflatoxin content of aflatoxin positive peanut meal samples.

When considering AEZs, the mean aflatoxin content in peanut meal sampled from Rainforest (224 µg/kg) was higher than the ones collected from Western High Plateau (146 µg/kg); all samples were positive for aflatoxin (*i.e.*, 100% occurrence) (Figure 2b). The average total aflatoxin content of positive maize from the Sahelian Zone was 2.40 µg/kg while positive samples from Western High Plateau contained 11.88 µg/kg (Figure 2a); averages for all samples were 1.35 µg/kg and 0.16 µg/kg for Western High Plateau and Sahelian, respectively. Occurrence of aflatoxin in maize was 10% (6/62) and 7% (1/15) in Western High Plateau and Sahelian respectively. Occurrence for broiler feeds was 92% (12/13) in Western High Plateau and 94% (16/17) in Rainforest. Occurrence in layer feeds was 85% (28/33) Western High Plateau and 80% (16/20) Rainforest.

**Figure 3:**
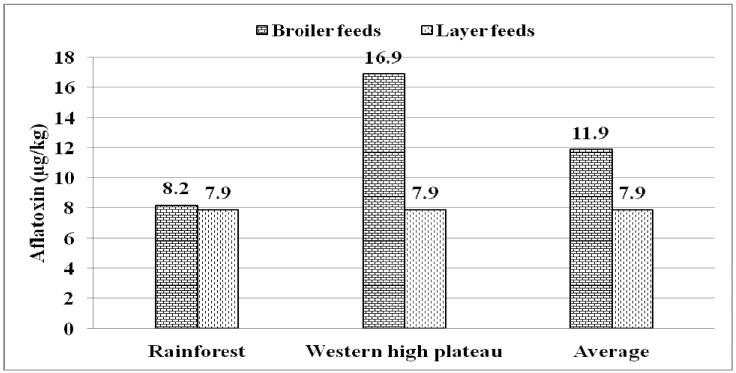
Average aflatoxin content of poultry feed mixtures collected in two agroecological zones in Cameroon.

The total aflatoxin content of layer feeds did not vary by AEZ, with an average of 7.9 µg/kg for both Rainforest and Western High Plateau (Figure 3). However, in broiler feeds the lowest aflatoxin content (8.2 µg/kg) was recorded in Rainforest AEZ as compared to Western High Plateau (16.9 µg/kg). The correlation between AEZs and total aflatoxins content was not significant for all the commodities. 

## 4. Discussion

The poultry industry in Cameroon produces 600,000 day old chickens weekly. The total production capacity of animal feed manufacturers including poultry feed is estimated to be over 150,000 tons per year, though current production is less than 100,000 tons [14] since import of chickens was banned. Fungal toxins including aflatoxin adversely affect poultry production and can also affect human health. While aflatoxins are prevalent between 40N and 40S latitudes globally, including in sub-Saharan Africa, there is no published information on the levels of aflatoxins in chicken feed in Cameroon. This study measured moisture content, which is a key factor influencing aflatoxin production, and aflatoxin levels in different poultry feeds from the three Cameroon AEZs with the highest number of poultry farms and maize production. The feeds exhibited high levels of aflatoxin accumulation, especially in peanut meal. The aflatoxin content of peanut meal exceeded 20 µg/kg (as high as 950 µg/kg), the legal limit in poultry feed formulation as recommended by Food and Drug Administration regulatory guidance for aflatoxin [15]. In this context, these toxins are a threat to the poultry industry and consumers in Cameroon. 

Favorable temperature and water activity which is an intrinsic parameter for the moisture content are crucial for mycotoxigenic fungi and mycotoxin production. In general, the extreme temperature and drought in relevant countries may make crops more liable to aflatoxins, like already experienced in Kenya during recent years [16]. Overall, the climatic conditions in tropical region are favorable for fungal development with high relative humidity [17,18], high temperature and moisture content [19,20] and little aeration [21], all conditions that accelerate fungal and mycotoxin development. The above mentioned climatic and environmental conditions are highly favorable for the propagation of fungi, especially the genera *Aspergillus*, *Penicillium* and *Rhizopus* that produce and release spores [22]. Independent to the AEZ, the moisture content of maize ranged from 11.7% to 17.7% with an average of 14.1% (Table 1). This is consistent with the finding of Kaaya and Kyamuhangire [23] who reported a mean moisture content of maize in three AEZs of Uganda around 13%. According to Mogan and Lacey [24], moisture content ≤15% is within the safe storage level for maize. However, molds were reported to grow over the temperature range of 10–40 °C and above 70% equilibrium relative humidity [25,26]. Some fungi were even reported by Lacey [27] and Thomson and Henke [28] to be capable of growing on a dry surface and on feeds containing 12%–13% moisture. The moisture content of maize (14.1%), broiler feeds (11.3%) and layer feeds (12.7%) recorded in our study fall within this range and can be suitable for fungal growth and toxin production if pH and relative humidity conditions are favorable. The moisture content of broiler feeds collected in Western High Plateau were higher compared to the Rainforest AEZ. This may be explained by the hot climate and high relative humidity (>80%) in the Western High Plateau as compared to conditions in the Rainforest AEZ [1].

This study revealed that approximately 9% of maize used in poultry feeds formulations in Cameroon is contaminated by aflatoxin with concentrations ranging from 2 to 42 µg/kg with an average of 1.0 µg/kg (Table 2). This average was lower compared to the results of Rodrigues *et al.* [2] who reported that between February and October 2009 about 94% of maize in Nigeria which borders Cameroon were contaminated by aflatoxin with an average concentration of 80.3 µg/kg. Our results also contrast with the findings of Kaaya and Kyamuhangire [23] who recorded 88%, 78% and 69% of maize contaminated by aflatoxin with an average concentration of 30, 22 and 12.8 µg/kg respectively in Mid-Altitude moist, Mid-Altitude dry and Highland zone in Uganda. In the present study, the aflatoxin content of peanut meal ranged from 39 to 950 µg/kg with an average of 161.4 µg/kg (Table 2). These results are lower compared to the findings of Abdelhamid [29] who recorded the average aflatoxin concentration of 400 µg/kg in peanut meal in Egypt. 

Most of the poultry feeds (87%) were positive for aflatoxin, and broiler feeds showed twice the levels of aflatoxin (11.1 µg/kg) compared to layer feeds (6.6 µg/kg), however the levels were not significant. Higher levels of aflatoxin in broiler feed could be explained by the fact that protein requirements of broilers chickens are higher than the needs of layers, and to meet the requirements, farmers use large quantities (up to 20%) of peanut meal in the formulation as cheapest proteins sources ingredient which is more susceptible to aflatoxins contamination. The present aflatoxin occurrence in poultry feeds 93% and 83% in broiler feeds and layer feeds respectively (averages 6.6–11.1 µg/kg) are very high compared to the 24% and 26% of poultry feeds which tested positive with an average concentration of 7 and 2 µg/kg reported in North and South America respectively between 2009 and 2011 [30]. 

Aflatoxin concentration in broiler feed was found to be highest in Western High Plateau and lowest in Rainforest agroecological zone. The environmental conditions, especially temperature and relative humidity/moisture prevailing in the Western High Plateau, may be responsible for this trend. The fact that most of poultry feeds from the Western High Plateau region (accounting for 60% of the poultry production in Cameroon) were positive to aflatoxin reveals that poultry production is likely to compromised [3] and poultry meat and eggs may be contaminated by aflatoxins [31,32]. 

## 5. Conclusion

Aflatoxin concentration of broiler feed was found to be highest in Western High Plateau, while layer feeds were the same. Environmental conditions in particular, high temperature and relative humidity/moisture, facilitate fungal growth and mycotoxin production. Peanut meal was the preferred substrate for fungal growth and exhibited the highest contamination level. Inappropriate storage conditions could be implicated in the fungal growth and aflatoxin production in feedstuffs and poultry feeds in Cameroon. There is a great need to develop practical and cost-effective methods of preventing mold growth and detoxifying poultry feed containing aflatoxin. 
